# Experimental Investigation on Interfacial Defect Criticality of FRP-Confined Concrete Columns

**DOI:** 10.3390/s19030468

**Published:** 2019-01-24

**Authors:** Renyuan Qin, Denvid Lau, Lik-ho Tam, Tiejun Liu, Dujian Zou, Ao Zhou

**Affiliations:** 1 Department of Architecture and Civil Engineering, City University of Hong Kong, Kowloon, Hong Kong, China; renyuaqin2-c@my.cityu.edu.hk (R.Q.); denvid.lau@cityu.edu.hk (D.L.); 2 School of Transportation Science and Engineering, Beihang University, 37 Xueyuan Road, Beijing 100191, China; leo_tam@buaa.edu.cn; 3 School of Civil and Environmental Engineering, Harbin Institute of Technology, Shenzhen, Shenzhen 518055, China; liutiejun@hit.edu.cn (T.L.); zoudujian@163.com (D.Z.)

**Keywords:** FRP, confinement, interfacial defect, stress–strain model

## Abstract

Defects between fiber reinforced polymer (FRP) and repaired concrete components may easily come out due to misoperation during manufacturing, environmental deterioration, or impact from external load during service life. The defects may cause a degraded structure performance and even the unexpected structural failure. Different non-destructive techniques (NDTs) and sensors have been developed to assess the defects in FRP bonded system. The information of linking up the detected defects by NDTs and repair schemes is needed by assessing the criticality of detected defects. In this study, FRP confined concrete columns with interfacial defects were experimentally tested to determine the interfacial defect criticality on structural performance. It is found that interfacial defect can reduce the FRP confinement effectiveness, and ultimate strength and its corresponding strain of column deteriorate significantly if the interfacial defect area is larger than 50% of total confinement area. Meanwhile, proposed analytical model considering the defect ratio is validated for the prediction of stress–strain behavior of FRP confined columns. The evaluation of defect criticality could be made by comparing predicted stress–strain behavior with the original design to determine corresponding maintenance strategies.

## 1. Introduction

Strengthening deteriorated concrete components using fiber reinforced polymer (FRP) has been validated to achieve the service extension for concrete structures. The maintenance schemes using FRP have been successfully applied for concrete beams, columns, slabs, and even timber structures [1,2,3,4,5,6]. For the application of FRP strengthened beams or columns, special attention still needs to be paid to identify the defects in FRP bonded system that may cause the deterioration of structural performance. Defects in FRP strengthened concrete structures could be induced from manufacture process, environmental deterioration, or impact from external load [7,8,9,10,11]. To detect the defects in FRP-concrete system, several non-destructive techniques and sensors have been developed to quantify the defect within the systems—such as acoustic-laser technique, optic-electronic sensors, laser-reflection technique, etc.—which can evaluate the material heterogeneity or defects through the measurement of vibrational frequency response for the materials [12,13,14,15,16,17,18]. After inspection, it is critical to evaluate the effect of detected defect, i.e., whether it can be merely neglected or significantly reduce the structural performance, so as to provide guidance on the repair schemes for the FRP reinforced concrete structures [19,20]. For FRP externally bonded concrete beams, it is reported that the defect size and location can significantly influence the criticality of corresponding defect on the structural performance of FRP bonded beam [7]. Meanwhile, two-sided effect can be found when there is interfacial defect between FRP and concrete substrate. The bond strength and fracture energy are found to be decreased with large defects due to the material damage. However, the small defects (within 1 mm of width) may benefit the interfacial bonding because of stress redistribution for FRP externally bonded beams [10]. Moreover, the experimental studies on FRP bonded beams with defects have also reported that the effect of defects within half of the FRP width on fracture toughness is negligible for FRP bonded concrete beams. However, most of the studies focus on the FRP bonded flexural members, and few results on the defect criticality of FRP confined concrete columns are reported [21,22,23,24]. Notably, defects in FRP bonded concrete beams can propagate rapidly into a plate end interfacial debonding and intermediate flexural crack induced interfacial debonding, which results in global failure of the structure, while defects in FRP confined columns can influence the confinement effectiveness of FRP and result in weakening of confined structures [25,26,27]. Due to defects in FRP confined concrete column, the shear stress cannot transfer effectively from FRP to concrete at localized defect region, and confinement effectiveness can be affected by stress redistribution [25]. Hence, it is important to evaluate the criticality of defects in FRP confined columns.

The interfacial defects between FRP and concrete, commonly identified in FRP bonded concrete systems, cause shear stress concentration and stress redistribution and result in debonding at corresponding cross-section of columns [28,29,30]. As the in-plane tensile stiffness of FRP is much higher than out-of-plane flexural stiffness, the interfacial defects tend to propagate in the hoop direction rather than axial direction when the FRP confined concrete columns are under compression. Hence, it is reasonable to consider the FRP confined columns with interfacial defects as a combination of partially FRP confined columns with bonding at intact region and FRP confined concrete columns without bonding, i.e., concrete-filled FRP tubes, at the defect region [31,32,33]. According to the previous studies, the effects of interfacial bonding can result in different confinement effectiveness coefficients and strain enhancement factors for FRP confined columns [34,35,36]. By adopting and combining the predictive models of partially FRP confined system at the intact layer and concrete-filled FRP tubes at defect layer considering the corresponding confinement effective coefficient and strain enhancement factor, the effect of interfacial defects on mechanical response of FRP confined columns becomes predictable.

The objective of this study is to investigate criticality of interfacial defect and its effect on the structural behavior of FRP confined columns. The FRP confined column with interfacial defects are made with ring-like defects with different height ratios of column height. This design is made by considering the extreme case that the interfacial defects will rapidly propagate and cause the debonding of entire cross-section at defect layer, due to the shear stress concentration and stress redistribution at corresponding defect layer. Moreover, the in-plane tensile stiffness of FRP is much higher than out-of-plane flexural stiffness, the interfacial defects have limited effects on other area out of the cross-sections where the defects are located at. Hence, ring-like defects were designed in this study to represent this extreme case that the entire defect layers lose the interfacial bonding. The uniaxial compressive tests are conducted to obtain the structural response and capture the failure modes of samples with different interfacial defect sizes. Furthermore, the predicted model on the structural response of FRP confined columns is proposed and verified according to experiments, and evaluations can be made based on the predicted results to determine the criticality of the defects and to decide whether an FRP strengthened member needs to be repaired. It is envisioned that the experimental findings and predicted model can provide some fundamental insights for the development of design guideline in consideration the criticality of interfacial defects and enable precise maintenance strategies for detected defects.

## 2. Materials and Methods

### 2.1. FRP Confined Columns and Defects Arrangment

The mix design of concrete used in this research for all the samples is provided in Table 1. The diameters of aggregates used for mixing concrete were ranged from 0 to 10 mm, and the slump of concrete was tested as 60 ± 10 mm according to ASTM C143. The concrete cubes were cast with the dimensions of 150 × 150 × 150 mm to test the compressive strength. For the concrete cylinders, the diameter and height of cylinder are 150 mm and 300 mm, respectively. All concrete samples were kept under the temperature of 23 °C for 28 days in water environment.

**Figure 1:**
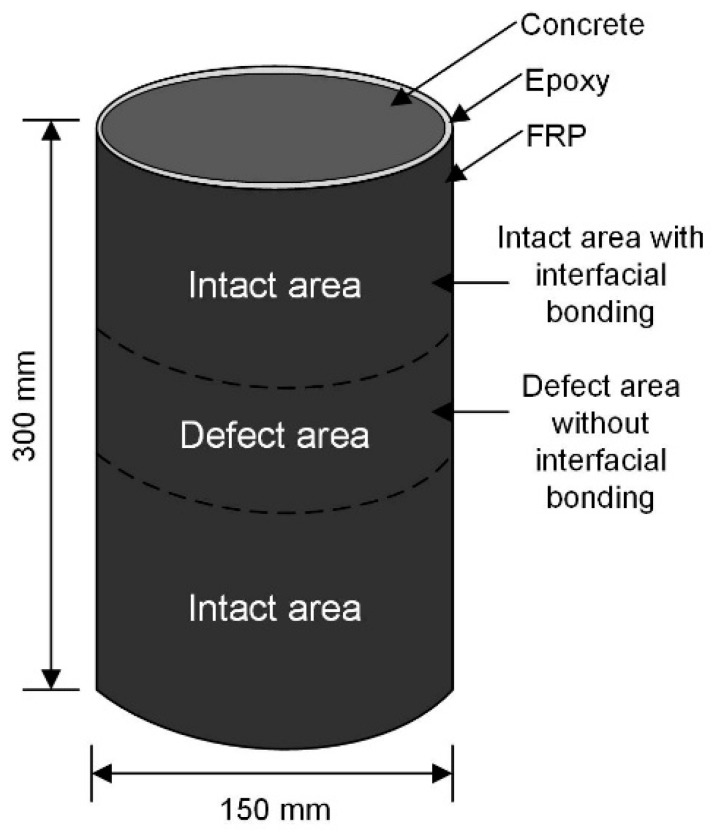
FRP confined concrete columns with interfacial defects.

The unidirectional carbon FRP was used as confinement material. The density and thickness of FRP are given as 1.60 g/cm^2^ and 0.167 mm. In order to obtain the tensile property of tensile FRP in longitudinal direction, tensile tests on FRP coupon were conducted according to ASTM D3039. The surface preparation of concrete cylinders by polishing and cleaning was made before bonding to FRP, which is critical to ensure the quality and reliability of bonding between concrete and FRP. The Teflon tapes were then bonded to the concrete at designed defect location, which can prevent the bonding between epoxy adhesive and concrete surface. It is noticed that the Teflon tape is inactive for the epoxy, preventing from further bonding between tape and epoxy, so that the interfacial defects is fabricated for FRP confined column samples. The heights of defects were designed as 30, 60, 120, 180, 240, and 300 mm, which were in the ratios of 10, 20, 40, 80, and 100%, respectively, to the height of column sample. A schematic diagram showing the dimensions of samples and arrangement of defects is shown in Figure 1. In total 16 concrete columns were tested including one group of unconfined samples and one group of fully confined samples without any interfacial defects. Table 2 shows the details of tested samples in terms of designed interfacial defects. Wet wrapping approach was adopted in this research with Sika 300 epoxy, which is one of the most widely used adhesive in construction industry in bonding FRP to concrete. A 150 mm overlap is adopted for the FRP confined samples, which is sufficient for the development of bond strength by epoxy. After fabrication of FRP confinement, a curing period of seven days passed before conducting further tests.

### 2.2. Test Instrumentation

The compressive test was firstly conducted for concrete cubes to quality mechanical properties of concrete according to BS EN 12390. Three unidirectional single layer CFRP coupons were tested according to ASTM standards to capture tensile property and rupture strain of FRP.

**Figure 2:**
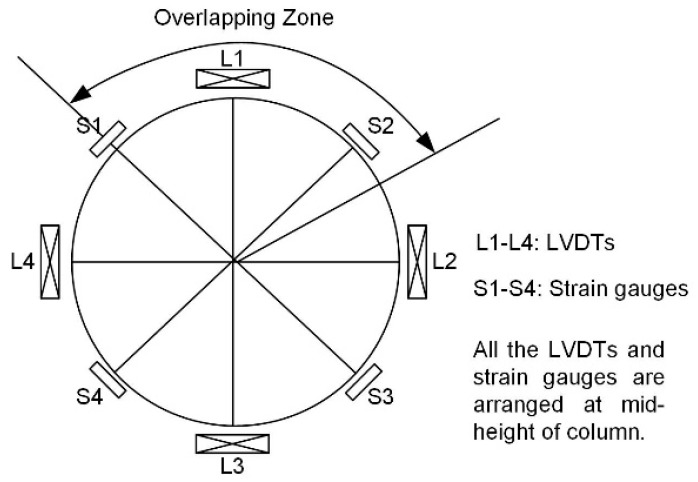
Arrangements of the LVDTs and strain gauges on the FRP confined columns.

**Figure 3:**
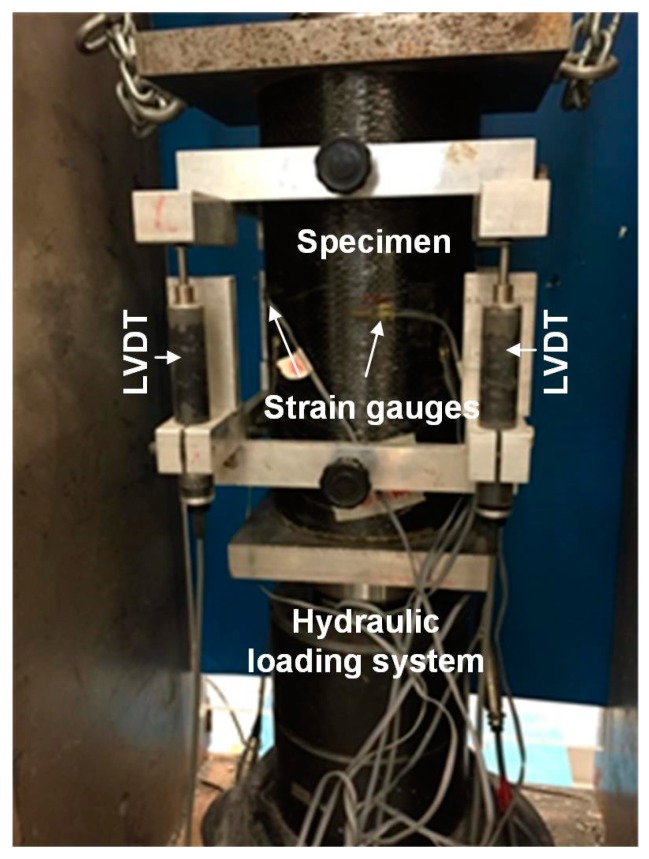
Experimental set-up for compressive test on FRP confined columns.

The uniaxial compressive tests were then conducted for FRP confined concrete columns. Four linear variable differential transformers (LVDTs) were arranged surrounding the circular sample and mounted on the aluminum frame fixing on the concrete. Meanwhile, four strain gauges were horizontally arranged at the mid-height of sample to capture the lateral strain. A schematic diagram of arrangement of LVDTs and strain gauges are shown in Figure 2. The compressive tests were conducted by the material test system with the capacity of 3000 kN under displacement control with a rate of 0.4 mm/min. The experimental set-up with main equipment is presented in Figure 3.

## 3. Results

The compressive strength and elastic modulus of concrete were tested as 43.7 MPa and 31.3 GPa, respectively according to the concrete cube test. The average ultimate strength and elastic modulus of FRP were determined as 3.69 GPa and 234 GPa, respectively, and the strain of FRP coupon was tested as 1.58% at rupture.

### 3.1. Stress–Strain Behavior

**Figure 4:**
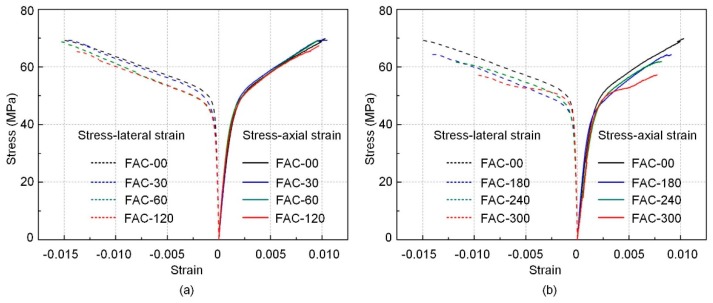
The stress–strain relationship of FRP confined concrete columns with interfacial defect size of (**a**) 10, 20, and 40%; and (**b**) 60, 80, and 100% to total confinement area compared to intact sample.

The effect of interfacial defects on the compressive performance of FRP confined column can be observed by the stress–strain response of specimen with and without interfacial defects. The stress–strain responses of all tested sample are described in Figure 4. In this study, after comparing the results from LVDTs and strain gauges, the data from LVDTs were used to calculate the axial strain and data from strain gauges to calculate the lateral strain, as the data from LVDTs are more accurate than those from vertical strain gauges, since they are not easily affected by localized post cracking of concrete and they can represent the structural response of FRP confined concrete column better. The experimental results of tested specimens are summarized in Table 3, in which *f*_co_ and *f*_cu_ are the ultimate strength of unconfined and confined concrete column, and *ε*_co_ and *ε*_cu_ are the strain at peak stress for plain concrete and confined columns, respectively.

As presented in Figure 4, the stress–strain response for specimens confined with FRP with or without interfacial defects can typically be divided into three portions. The first stage is a linear ascending segment where the FRP concrete specimens are in the elastic stage. A transition segment follows the linear ascending segment can be found then. In this period, the microcracks in the concrete are generated and propagate with the increasing load, and FRP jackets start to carry load gradually with the stress transferred from concrete through interface and a smooth inflection could be found in the stress–strain curves. The third portion is another ascending segment in which hoop dilation of specimen due to increasing load is carried by FRP. The termination of the third segment determined the by the failure of FRP confinement. The corresponding energy absorption, which is an important criterion for the effectiveness of the confinement, can be determined according to the stress–strain curves using the expression Σ(d*σ*)(d*ε*) [37]. The corresponding results are shown in Table 3.

The stress–strain responses of FRP confined samples with various sizes of interfacial defects show different behaviors. This difference comes from the starting point of the transition segment and stiffness of the strain-hardening portion, especially for specimens with the interfacial defects area larger than 50% of the total FRP confinement area. As aforementioned, the FRP confined concrete column with interfacial defects could be regarded as the combination of partially FRP confined concrete column at intact area and FRP confined concrete column without interfacial bonding at defect area. According to the previous research on partially FRP confined system, when the volume fraction of FRP jacket is less than 50%, a monotonic strain-hardening behavior cannot be achieved in the third segment of the stress–strain curves [33]. The strength enhancement is not significant when the volumetric ratio of FRP jacket is less than 50% [32]. Similar phenomena can be found from presented tests. When the defect ratio is larger than 50%, third ascending segment shows significant difference compared with that of FRP confined concrete columns without interfacial defects. Because the defect region is still confined by FRP with a reduced confinement stiffness, such confinement without interfacial bonding can still provide enhancement in terms of strength by FRP. Therefore, the decreasing trend is not observed in the third segment of stress–strain curves. However, the significant reduction in terms of ultimate strength can be found when the area of interfacial defect larger than 50% of total confinement area. Specifically, for the specimens with the interfacial defect size of 60, 80, and 100% of total confinement area, the ultimate strengths are 64.27, 61.39, and 58.36 MPa, respectively, which are reduced by 8.1, 12.3, and 16.5% compared to the ultimate strength of samples without defects. For the specimens with the interfacial defect size of 10, 20, and 40% of total confinement area, the ultimate strengths are 69.22, 69.28, and 67.71 MPa, respectively, which are just reduced by 2.4, 2.3, and 3.1% compared to the that of specimens without defects. Similarly, the ultimate axial strains also reduce significantly when the defect ratio is larger than 50%. For specimens with the interfacial defect size of 60, 80, and 100% of total confinement area, the ultimate strains are 0.0091, 0.0082, and 0.0081, respectively, which are reduced by 13.3, 21.9, and 22.8% compared with that of intact samples. For the FRP confined columns with the interfacial defect size lower than 50% of total confinement area, no significant reduction can be found for ultimate strain.

For stress–strain behavior in hoop direction shown in Figure 4. When the size of interfacial defect is smaller than 50% of total confinement area, the lateral strain is close to FRP rupture strain, which indicates that the failure happened at mid-height due to the rupture of FRP. However, when the size of interfacial defect is larger than 50% of confinement area, the measured lateral strain at mid-height level decrease significantly compared to the rupture strain of FRP. This reduction should be due to that with the increasing size of interfacial defects, stress concentration is more severe at edge of defect area, where the failure would initiate at the edge of the defect area rather than at mid-height level. Hence, the measured lateral strain at failure is less than the rupture strain of FRP determined in the coupon test. The results from the variation of lateral strain can also explain the reduction in terms of ultimate strength. For the specimens with defect size less than 50% of total confinement area, the measured ultimate lateral strain for the FRP is close to its rupture strain in the coupon test, indicating that the confining pressure provided by the FRP jacket is similar to that in the samples without interfacial defects. The calculated ultimate stresses of FRP according to the lateral strain are 3.65, 3.58, and 3.35 GPa for FAC-30, FAC-60, and FAC-120, respectively, which are close to the tensile strength of FRP in the coupon test. These results indicate that in these samples, the confining pressure is effectively developed in FRP jacket. With a similar confinement effectiveness provided by FRP, the ultimate strength and its corresponding strain is considered as similar according to design codes for FRP confined concrete column, as shown in Figure 4a. While for specimens FAC-180, FAC-240, and FAC-300, the ultimate stresses of FRP are determined as 3.00, 2.94, and 2.38 GPa. These data indicate that confining pressure has not carried by FRP jacket effectively before the failure of the entire composite system, resulting in a reduction in ultimate strength of FRP confined concrete columns.

### 3.2. Failure Modes

**Figure 5:**
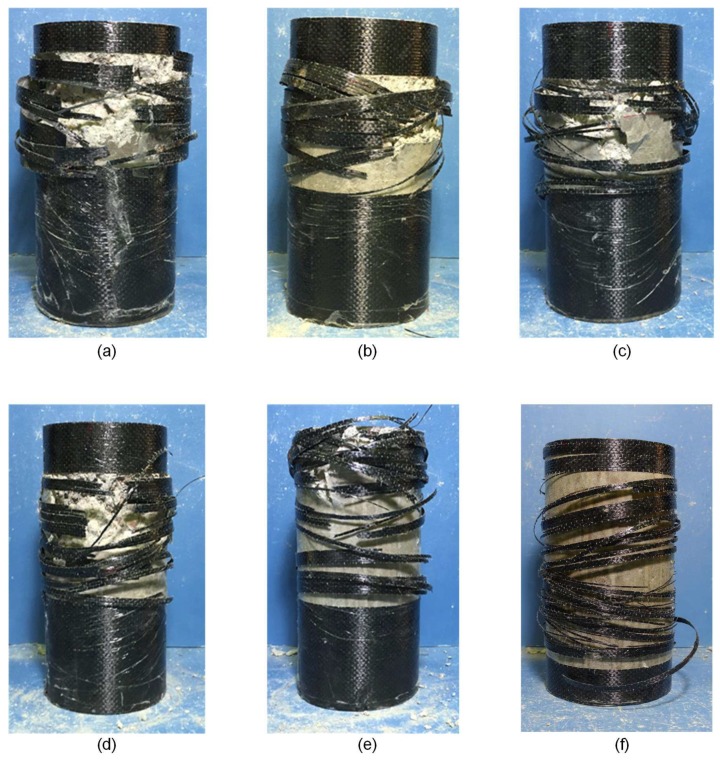
The failure photos of specimen with different sizes of interfacial defect: (**a**) FAC-30; (**b**) FAC-60; (**c**) FAC-120; (**d**) FAC-180; (**e**) FAC-240; and (**f**) FAC-300. With the increasing of interfacial defect area, FRP jacket fails into small pieces. The concrete in the non-bonded region cracks into several bulks without further crushing, while the concrete in the region with the interfacial bonding of FRP is broken into small pieces and even ashes.

The photos capturing the failure modes of tested specimens are shown in Figure 5. The failure of FRP confined columns is mainly constituted by fracture of FRP confinement and concrete crushing. For the failure process of these specimens, the rupture of epoxy occurs firstly followed by FRP failure. For the specimens with the interfacial defect smaller than 20% of total confinement area, the FRP confinement failed around mid-height of cylinder and outside of overlapping zone. For the specimens with interfacial defect larger than 20% of total confinement area, the rupture of FRP tend to occur firstly near the edge of interfacial defect because of stress concentration, followed by the rupture of FRP within area with the interfacial defect. For the specimens with interfacial defects, the failure of FRP mainly occurs at the region with interfacial defects, while the bonded region between FRP and concrete is usually remained as intact and fails lastly. Moreover, it is found that the concrete in the non-bonded region cracks into several bulks without further crushing, while the concrete in the region with the interfacial bonding of FRP is broken into small pieces and even ashes, which indicates that the confinement effectiveness provided by FRP without interfacial bonding is much lower than that with interfacial bonding. Such evolution of observed damage with the increase of defect sized can be observed in Figure 5 as well. Hence, an analytical model considering this variation in terms of FRP confinement effectiveness coefficient for defect and intact area would be capable to describe structural behavior of FRP confined concrete column with interfacial defect.

## 4. Discussions

### 4.1. Ultimate Performance of FRP Confined Column with Defect

The compressive behavior of columns is determined by *f*_cu_ and *ε*_cu_ of FRP confined column specimens. The values of *f*_cu_ and *ε*_cu_ for all columns under compression are summarized in Table 3.

**Figure 6:**
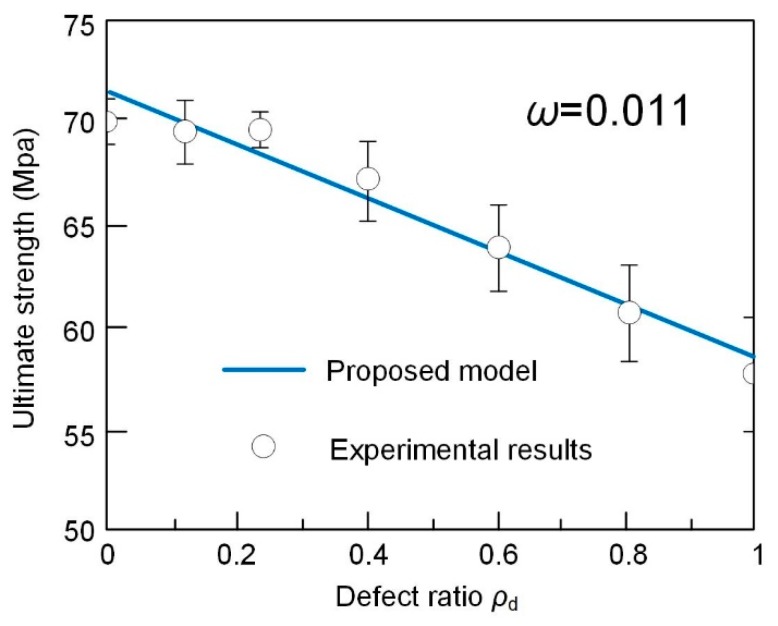
Comparation between ultimate strength predicted by proposed model and tested results.

According to the literature on the prediction of *f*_cu_, many models have been developed based on Richart’s model, which can be expressed as [38]

(1) $$\frac{f_{\mathrm{cu}}}{f_{\mathrm{co}}}=1+k_{1}\frac{f_{l}}{f_{\mathrm{co}}}$$

where *f*_l_ is confinement stress provided by FRP, which is expressed as

(2) $$f_{l}=\frac{2E_{\mathrm{frp}}\varepsilon_{\mathrm{rup}}t_{f}}{D}$$

in which *E*_frp_, *ε*_rup_, and *t*_f_ are the Young’s Modulus, rupture strain and nominal thickness of FRP, respectively. Moreover, by considering intact area as partially FRP confined system, this pressure could be further expressed as [39]

(3) $$f_{l}^{\prime }=\frac{2E_{\mathrm{frp}}\varepsilon_{\mathrm{rup}}t_{f}}{D}\frac{w}{w+s}$$

where *w* and *s* are the width of FRP band and distance between each two bands, respectively, and D is the diameter of specimen. Moreover, there is a vertical coefficient *k*_v_ considering the variation of confined and unconfined zone. Since the entire concrete column is confined by FRP, vertical confinement coefficient is not taken for this model although there are the interfacial defects. It can be seen from to Equation (1), the main difference of FRP confined concrete column with and without interfacial bonding comes from the *k*_1_. According to the existing studies on the confinement effectiveness coefficient considering the effect of interfacial bonding, *k*_1_ is taken as 3.5 for FRP confined concrete column with bonding and 2 for FRP confined concrete column without interfacial bonding [40]. Eventually, the ultimate strength of FRP confined column with interfacial defect is calculated by

(4) $$\frac{f_{\mathrm{cu}}}{f_{\mathrm{co}}}=1+3.5\frac{f_{l}^{\prime }}{f_{\mathrm{co}}}+2\rho_{d}\frac{f_{l}}{f_{\mathrm{co}}}$$

where $\rho_{d}$ is the defect ratio. The performance of predicted value and experimental ones are plotted in Figure 6. good agreements between predicted results and experimental values can be found. By defining the error index, ω, as the summation of deviation of experimental results and predicted value over the summation of experimental results, the error index is calculated as 0.011, which validates the accuracy of proposed model in prediction of the ultimate strength for samples with different defect areas. It should be noticed that when $\rho_{d}$ equals to 0, which represents the case of no interfacial defect, Equation (4) is exactly with the same form of models in prediction of normal FRP confined column. When $\rho_{d}$ equals to 1, the equation is with the same form of model in prediction of concrete filled FRP tube. Hence, the proposed model is also available for predicting the extreme condition.

The corresponding axial strain at ultimate strength, *ε*_cu_, has following relationship with the ultimate strength, *f*_cu_, according to Richart et al. [38]

(5) $$\frac{\varepsilon_{\mathrm{cu}}}{\varepsilon_{\mathrm{co}}}=1+5\left(\frac{f_{\mathrm{cu}}}{f_{\mathrm{co}}}-1\right)$$

According to the Equations (1)–(4), the predictive model for axial strain at ultimate strength for columns with interfacial defects can be expressed as following equation after the calibration of test results [32].

(6) $$\frac{\varepsilon_{\mathrm{cu}}}{\varepsilon_{\mathrm{co}}}=1+17.5{\left(\frac{f_{l}^{\prime }}{f_{\mathrm{co}}}\right)}^{1.2}+10\rho_{d}\frac{f_{l}}{f_{\mathrm{co}}}$$

**Figure 7:**
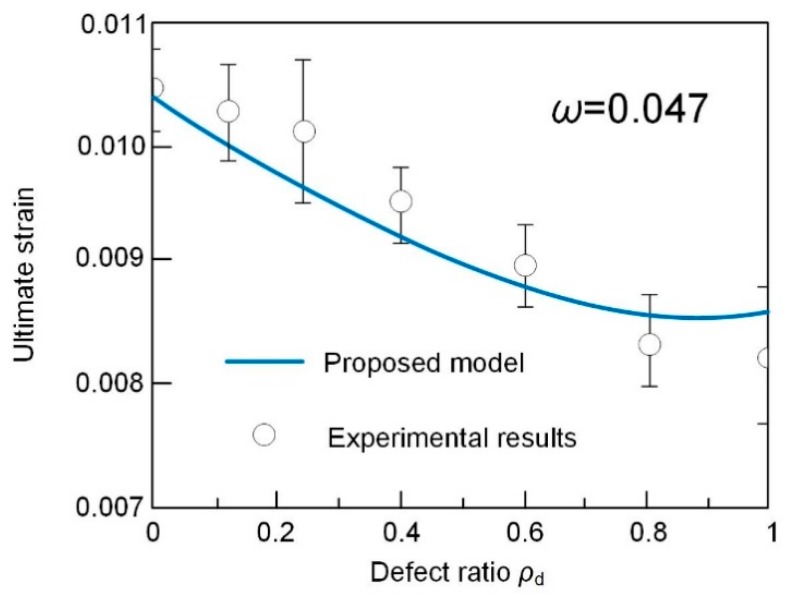
Comparation between ultimate strain from model prediction and tested results.

The performance of predicted value and test ones are presented in Figure 7. The error index is determined as 0.047, which validates the accuracy of proposed model in prediction the axial strain of specimens with different defect sizes.

### 4.2. Stress–Strain Model of FRP Confined Column with Interfacial Defects

The compressive behavior observed in experiments is of the typical form that has been reported in the literature in terms of stress–strain relationship, which consists a parabola initial segment followed by a linear segment, as shown in Figure 4. The slope of second linear segment reduces with the increase of defect size while a similar initial stiffness. Hence, a design-oriented model that has a clear definition of slope for the second linear segment is adopted in this study [35,41]. Moreover, this design-oriented model can make accurate prediction without calibrating any other parameters apart from material properties of FRP and concrete. Hence, it is considered as an approach that is more flexible for engineering practice.

**Figure 8:**
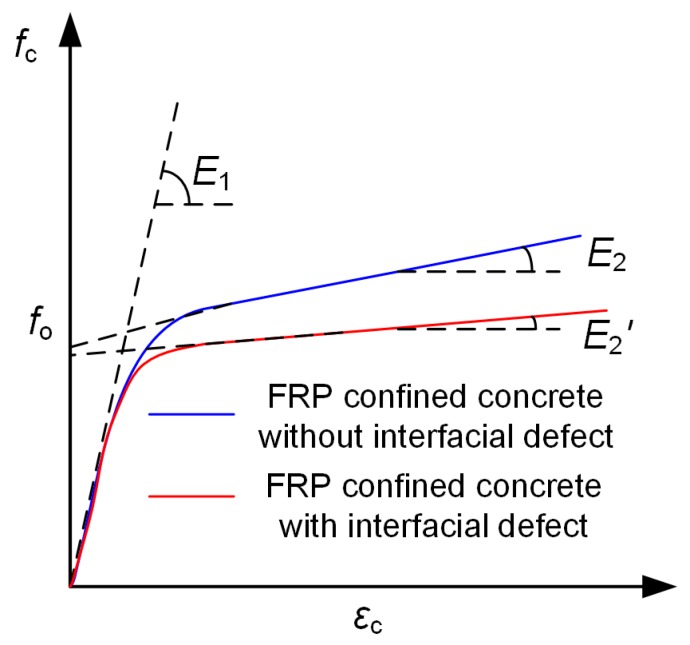
Schematic diagram showing the definition of parameters in used model [36].

The general equation describing stress–strain response for FRP confined column is expressed as [35]

(7) $$\sigma_{c}=E_{c}\varepsilon_{c}-\frac{{\left(E_{c}-E_{2}\right)}^{2}}{4f_{o}}\varepsilon_{c}^{2}\;\mathrm{when}\;0\leq \varepsilon_{c}\leq \varepsilon_{t}$$

and

(8) $$\sigma_{c}=f_{o}+E_{2}\varepsilon_{c}\;\mathrm{when}\;\varepsilon_{t}\leq \varepsilon_{c}\leq \varepsilon_{\mathrm{cu}}$$

where *f*_o_ is a parameter determining the intercept linear segment, and it is taken as *f*_co_ in this study, which is also widely adopted in literature [42,43]. *ε*_t_ is strain data point connecting first parabolic and second linear portion; *E*_2_ is slope of the second linear portion, which could be expressed as

(9) $$\varepsilon_{t}=\frac{2f_{o}}{\left(E_{c}-E_{2}\right)}$$

(10) $$E_{2}=\frac{f_{\mathrm{cu}}-f_{o}}{\varepsilon_{\mathrm{cu}}}$$

where *f*_cu_ and *ε*_cu_ can be calculated based on the Equations (4) and (6). The elastic modulus is taken as $4730\sqrt{f_{\mathrm{co}}}$ for concrete materials. The meaning of each parameter is also shown in Figure 8.

**Figure 9:**
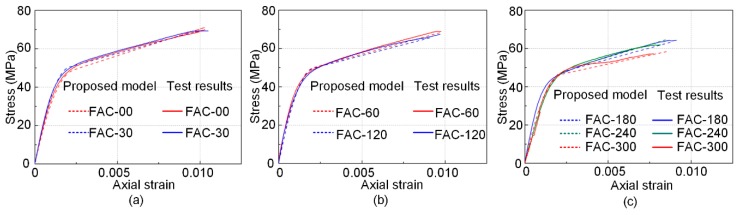
Comparison between the stress–strain relationships predicted by proposed model and experimental results. (**a**) samples FAC-00 and FAC-30; (**b**) samples FAC-60 and FAC-120; and (**c**) samples FAC-180, FAC-240 and FAC-300.

The stress–strain curves can then be generated based on Equations (7)–(10). The stress–strain curves generated from predicted model and experimental results are plotted in Figure 9 with good agreements for all the specimens. When the defect size is larger than 50% of total area of confinement, the ultimate strength and strain ratio is significantly reduced from the design level. In the engineering practice, the defects in FRP confined concrete systems could be encountered in different sizes and shapes, such as localized defects. From the viewpoint of safety, it is recommended that when the localized interfacial defects are detected, the corresponding cross-section could be regarded as interfacial debonding; and reduction of ultimate strength, strain ratio, and stress–strain relationship should be considered for the evaluation of the criticality of defect using proposed model. The corresponding repair scheme such as resin injection, ply replacement, or overlapping FRP sheets can be determined based on the predicted reduction compared to the originally designed performance. Moreover, the methods and models shown in this study can provide insights on the future study of evaluation scheme on partial and nonuniform debonding damages by considering different reduction in terms of FRP confinement effectiveness coefficient to further development an evolution scheme for interfacial defects with different forms.

## 5. Conclusions

The criticality of interfacial defect in FRP confined concrete column system was investigated experimentally in this study. The ultimate strength and ultimate strain of FRP confined concrete column are slightly reduced within 5% when the area of interfacial defects is smaller than 50% of total confinement area. However, a significant reduction is observed when the size of interfacial defect is larger than 50% of total confinement area. For specimens with the interfacial defect ratio of 60, 80, and 100% to total confinement area, the ultimate strength is reduced by 8.1, 12.3, and 15.6%, respectively, and the ultimate strain ratio is reduced by 13.3, 21.9, and 22.8%, respectively, compared to those of FRP confined concrete sample without interfacial defect. Such results indicate that the FRP confinement effectiveness is significantly affected by interfacial debonding, and the load-bearing capacity as well as ductility of column needs to be re-evaluated when the interfacial defect between FRP and concrete is detected.

Based on experimental results, an analytical model is developed for prediction of ultimate behavior of FRP confined columns with interfacial defects. The predicted results are validated by experimental results in terms of peak stress and strain, where good agreements are achieved. The proposed model can be further adopted in existing design-orientated models for the evaluation of FRP confined columns with interfacial defects in terms of stress–strain response. The defect criticality could be determined by evaluating predicted stress–strain behavior and the original designed performance. The corresponding repair scheme such as epoxy resin injections, cement mortar overlays, fiber reinforcing mesh, and externally FRP confinement can be applied based on the detected defects as well as the designed performance of the structure in terms of load-bearing capacity and ductility [44]. The findings in this study can provide some fundamental insights on the development of evaluation scheme and maintenance strategy for the detected defects in FRP bonded concrete system.

## Figures and Tables

**Table 1:** Concrete mix design.

Cement (kg/m^3^)	Sand (kg/m^3^)	Aggregate (kg/m^3^)	Water/Cement Ratio	Density (kg/m^3^)
383	792	968	0.6	2374

**Table 2:** Summary of tested concrete column samples.

Specimen Name	No. of Samples	Defect Height	Percentage to Height
FAC-plain	2	Unstrengthened	--
FAC-00	2	Strengthened	--
FAC-30	2	30 mm	10%
FAC-60	2	60 mm	20%
FAC-120	2	120 mm	40%
FAC-180	2	180 mm	60%
FAC-240	2	240 mm	80%
FAC-300	2	300 mm	100%

**Table 3:** Summary of tested specimens with different sizes of defect.

Specimen	Defect Height to Column Height	Ultimate Strength (MPa)	Ultimate Axial Strain	Ultimate Lateral Strain	*f*_cu_/*f*_co_	*ε*_cu_/*ε*_co_	Energy Absorption (MJ/m^3^)
FAC-Plain	-	42.32	0.0021	-	-	-	
FAC-00	-	69.87	0.0105	0.0157	1.65	5.00	0.585
FAC-30	10%	69.22	0.0103	0.0156	1.64	4.90	0.563
FAC-60	20%	69.28	0.0102	0.0153	1.64	4.90	0.532
FAC-120	40%	67.71	0.0097	0.0141	1.59	4.62	0.521
FAC-180	60%	64.27	0.0091	0.0131	1.52	4.33	0.466
FAC-240	80%	61.39	0.0082	0.0126	1.45	3.90	0.399
FAC-300	100%	58.36	0.0081	0.0102	1.38	3.85	0.358
